# Identification of candidate genes for drought tolerance by whole-genome resequencing in maize

**DOI:** 10.1186/1471-2229-14-83

**Published:** 2014-04-01

**Authors:** Jie Xu, Yibing Yuan, Yunbi Xu, Gengyun Zhang, Xiaosen Guo, Fengkai Wu, Qi Wang, Tingzhao Rong, Guangtang Pan, Moju Cao, Qilin Tang, Shibin Gao, Yaxi Liu, Jing Wang, Hai Lan, Yanli Lu

**Affiliations:** 1 Maize Research Institute, Sichuan Agricultural University, Wenjiang 611130 Sichuan, China; 2 Key Laboratory of Biology and Genetic Improvement of Maize in Southwest Region, Ministry of Agriculture, Wenjiang 611130 Sichuan, China; 3 Institute of Crop Science, the National Key Facilities for Crop Genetic Resources and Improvement, Chinese Academy of Agricultural Sciences, Beijing 100081, China; 4 BGI-Shenzhen, Shenzhen 518083, China; 5 International Maize and Wheat Improvement Center (CIMMYT), El Batan 56130 Texcoco, Mexico; 6 Triticeae Research Institute, Sichuan Agricultural University, Wenjiang 611130 Sichuan, China

## Abstract

**Background:**

Drought stress is one of the major limiting factors for maize production. With the availability of maize B73 reference genome and whole-genome resequencing of 15 maize inbreds, common variants (CV) and clustering analyses were applied to identify non-synonymous SNPs (nsSNPs) and corresponding candidate genes for drought tolerance.

**Results:**

A total of 524 nsSNPs that were associated with 271 candidate genes involved in plant hormone regulation, carbohydrate and sugar metabolism, signaling molecules regulation, redox reaction and acclimation of photosynthesis to environment were detected by CV and cluster analyses. Most of the nsSNPs identified were clustered in bin 1.07 region that harbored six previously reported QTL with relatively high phenotypic variation explained for drought tolerance. Genes Ontology (GO) analysis of candidate genes revealed that there were 35 GO terms related to biotic stimulus and membrane-bounded organelle, showing significant differences between the candidate genes and the reference B73 background. Changes of expression level in these candidate genes for drought tolerance were detected using RNA sequencing for fertilized ovary, basal leaf meristem tissue and roots collected under drought stressed and well-watered conditions. The results indicated that 70% of candidate genes showed significantly expression changes under two water treatments and our strategies for mining candidate genes are feasible and relatively efficient.

**Conclusions:**

Our results successfully revealed candidate nsSNPs and associated genes for drought tolerance by comparative sequence analysis of 16 maize inbred lines. Both methods we applied were proved to be efficient for identifying candidate genes for complex traits through the next-generation sequencing technologies (NGS). These selected genes will not only facilitate understanding of genetic basis of drought stress response, but also accelerate genetic improvement through marker-assisted selection in maize.

## Background

Drought is one of the most important environmental stresses around the world [1]. The climate changes and increasing population pose serious challenges to crop improvement. It is believed that understanding of how plants respond to drought stress at the molecular level are useful for developing improved genotypes which would perform well under water-limited conditions [2]. Maize (*Zea mays* spp. *mays L*.), one of the most important food crops in the world, is very sensitive to water-deficiency, especially during flowering, pollination and embryo development [3].

Previous studies reaffirmed that drought tolerance is a complex trait controlled by many genes [4]. It is important to mine candidate genes and unravel molecular mechanisms in response to drought stress in maize, which would help accelerate genetic improvement through marker-assisted selection. So far, genetic studies using strategies such as quantitative trait locus (QTL) mapping, subtractive hybridization (SSH), Real Time-PCR and cDNA microarray technology, have been reported in maize [2,5-8]. However, QTL identified under a specific genetic background usually show relatively small effects or even cannot be detected under other genetic backgrounds [9], and several studies have been done to integrate the results from multiple independent QTL mapping experiments to unravel genetic factors underlying complex traits [9-11].

Despite the surfeit of mapping publications, only a few QTL have been identified to date at the gene level through map-based cloning due to the complexity of the maize genome [12], resulting in largely unknown mechanisms of drought response. The next-generation sequencing (NGS) technologies, which provide direct insight into the DNA variation, have been used for genome-wide sequencing (GWS), polymorphism detection and marker development, DNA methylation and histone modification, alternative splicing identification, gene expression analysis and DNA-protein interactions [13-15]. NGS has also become a vital choice for identifying candidate genes and variants underlying simple and even complex traits through linkage mapping, association mapping and other approaches [5]. A known QTL (*GW*5) associated with rice grain width was successfully identified using 209 K SNPs that were produced by whole-genome resequencing of a recombinant inbred line population [16]. Besides, transcriptome sequencing is also applied in transcriptional and post-transcriptional regulation analyses of genes under abiotic stress and global expression pattern analysis of complex genomes [17-20]. The transcriptome of maize reference genome B73 was studied using RNA-seq to compare gene expression in fertilized ovaries and basal leaf meristem tissues collected under drought-treated and well-watered conditions [17]. Moreover maize miRNAs regulating abiotic stress-associated processes and the gene networks were identified, and a gene model showing how they worked was proposed [20,21].

Finding and exploiting DNA sequence variation within a genome is of utmost importance for crop genetics and breeding. Thanks to the availability of whole-genome or transcriptome sequences in public databases and the recent advent of bioinformatics tools, mining genetic variation has become easier and more cost-effective. The objectives of this study are to 1) screen SNPs that play important roles in maize drought tolerance using genome-wide sequencing data; 2) identify corresponding candidate genes based on the identified nsSNPs and compare them with reported QTL for drought tolerance; 3) detect changes in expression level of these candidate genes using RNA-seq data from different maize tissues under two water treatments. The candidate genes could be the fundamental genetic resource for enhancement of maize drought tolerance, and their expression analysis and insight into molecular mechanisms would be helpful for molecular breeding towards improving abiotic stress adaptation.

## Results

### SNPs and their distribution in maize genome

Whole-genome resequencing was performed for 15 maize inbreds and a total of 4.6 billion (407 gigabases) sequence reads were aligned against the maize B73 reference genome using Short Oligonucleotide Alignment Program 2 (SOAP 2) [22], resulting in 85% of genome coverage on average. The detailed resequencing information was provided in Additional file 1: Table S1. A total of 6,385,011 SNPs with high quality were called from 15 maize inbreds and B73 reference genome. The number of SNPs was the most on chromosome 1 (2,511,910) and the least on chromosome 10 (1,205,225), accounting for 15.33% and 7.36% of the SNPs, respectively. SNP density varied among chromosomes, and chromosome 1 has the highest density with 8.34 SNPs per Kb and chromosome 5 has the lowest density with 7.29 SNPs per Kb (Table 1).

**Table 1:** Summary of SNPs and their distribution in different genomic regions

**Chr.**	**Total SNP**		**Exonic region**				**UTR**			**Splicing sites**^**e**^	**Intronic region**	**Promotor**	**Intergenic region**
	**Number**	**Density**^**a**^	**Nonsyn**	**Syn**	**Stop-gain**^**b**^	**Stop-loss**^**c**^	**5′-UTR**	**3′-UTR**	**5′-UTR/3′-UTR**^**d**^				
1	2,511,910	8.34	31,289	36,761	619	126	14,036	29,588	631	725	213,368	80,967	2,103,800
2	1,915,130	8.08	25,916	28,767	442	108	10,644	22,370	402	567	150,733	60,545	1,614,636
3	1,862,945	8.03	22,218	25,182	451	106	9,370	18,894	295	494	138,043	55,225	1,592,667
4	1,991,721	8.25	20,323	21,608	456	96	8,740	17,449	458	447	117,866	54,140	1,750,138
5	1,588,421	7.29	22,810	27,197	371	105	10,315	22,646	474	539	142,569	56,768	1,304,627
6	1,270,649	7.51	16,936	19,001	308	77	7,589	14,610	375	424	98,366	43,059	1,069,904
7	1,367,126	7.73	16,692	19,323	301	60	7,361	14,499	301	388	105,085	40,322	1,162,794
8	1,403,887	7.99	18,411	20,233	376	75	43,688	16,400	290	452	111,501	46,710	1,145,751
9	1,267,997	8.09	14,748	17,468	279	71	6,789	14,366	245	355	96,433	38,962	1,078,281
10	1,205,225	8.02	15,871	17,457	240	59	5,978	12,369	120	287	83,062	34,006	1,035,776
Total/Average	16,385,011	7.95	205,214	232,997	3,843	883	124,510	183,191	3,591	4,678	1,257,026	510,704	13,858,374
Percentage	-	-	1.25%	1.42%	0.02%	0.01%	0.76%	1.12%	0.02%	0.03%	7.67%	3.12%	84.58%

*Chr* chromosome. Nonsyn: nonsynonymous SNPs. *Syn* synonymous SNPs. ^a^Density: SNP number per Kb. ^b^ Stop-gain: changes an amino acid to a STOP codon. ^c^Stop-loss: the mutation results in the loss of a STOP codon. ^d^5′-UTR, 3′-UTR: SNPs located in 5′-UTR or 3′-UTR of different transcripts. ^e^Splicing sites: SNPs in different genomic regions refer to alternative splicing.

The distribution of SNPs across genomic regions was compared (Table 1). SNPs were most abundant in intergenic regions (84.58%), followed in an order of intronic, promotor, exonic, UTR and splicing site regions. Notably, more SNPs were located in 3’-UTR (1.12%) than in 5′-UTR (0.76%). Moreover, more SNPs were detected in the introns (7.67%) than exons (2.70%). For exonic regions, there were 232,997 synonymous SNPs, 205,214 nsSNPs, 3843 stop-gain and 883 stop-loss mutations. Synonymous SNPs were more abundant and the average non-synonymous to synonymous substitution ratio (Nonsyn/Syn ratio) was 0.42 in the exonic regions.

### Detection of nsSNPs and candidate genes using common variants (CV) analysis

**Figure 1:**
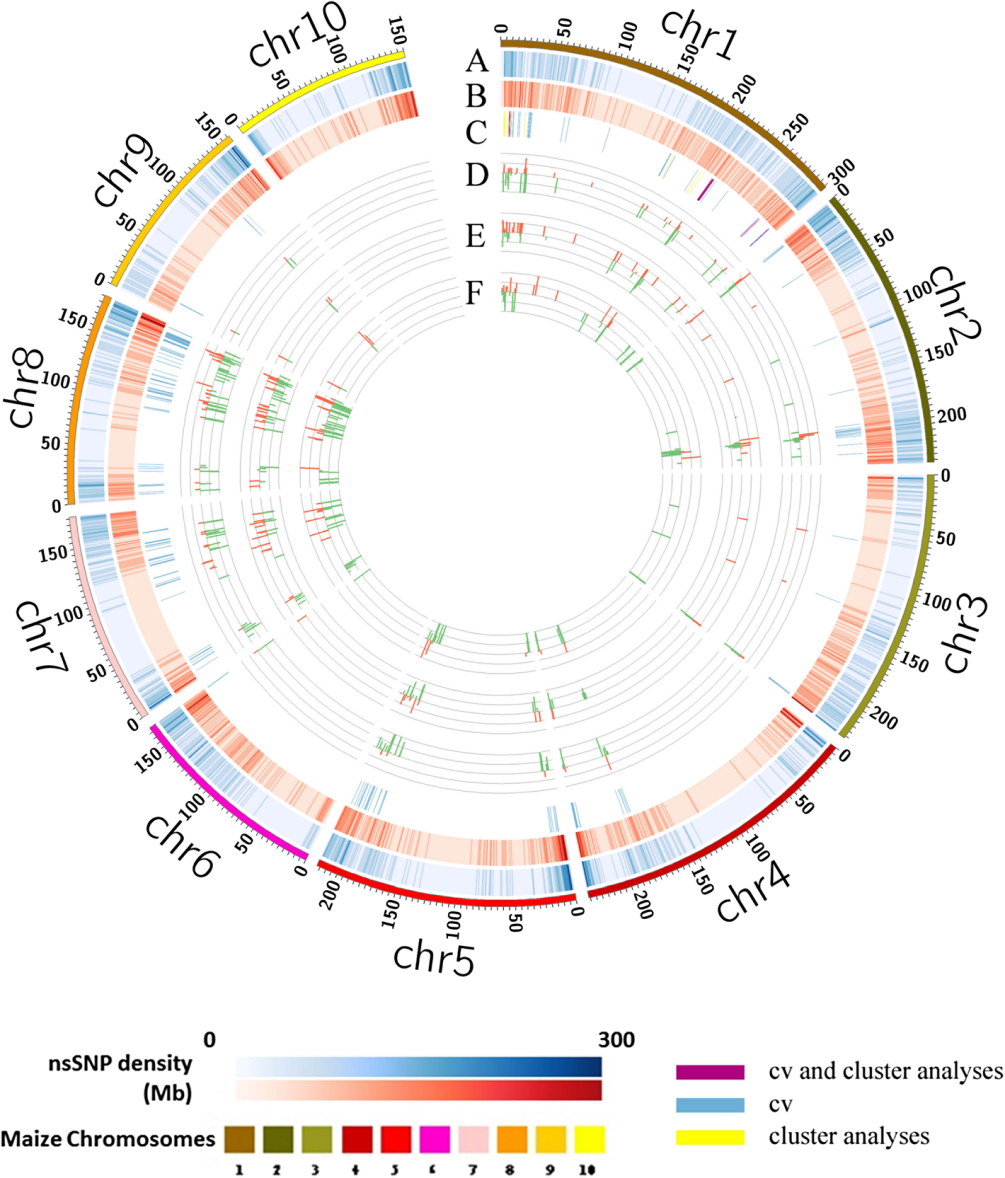
**Distribution of nsSNPs and associated genes on maize chromosomes.** Concentric circles showed aspects of the genome. Density of common nsSNPs identified in drought-tolerant maize inbreds **(A)** and in drought-sensitive maize inbreds **(B)**. Genome mapping of candidate nsSNPs identified by common variants method **(C)**. The fold change of expression level for candidate genes in ovaries **(D)**, leaves **(E)** and roots **(F)** under water-stressed conditions compared with well-watered conditions. For Figure C, different colors indicate different strategies as shown at the bottom of right corner. For Figures D, E and F, red and green bars represent up- and down- regulated expression, respectively.

ANNOVAR tool was applied to filter nsSNPs [23]. Three extremely drought-tolerant lines and three drought-sensitive lines were used to detect candidate nsSNPs [24-29]. There were 105,656 and 89,263 nsSNPs sharing the same variants within drought sensitive group and drought tolerant group, respectively. The variants distributed across genome regions showed different densities between the two groups (Figure 1A and B). There were more variants located in telomeric regions than in near centromere regions, which was in accordance with the distribution patterns of genes in maize [30]. Among the variants, 499 nsSNPs (0.24%) associated with 259 genes (266 transcripts) were different between the groups (Figure 1C). Chromosomes 1 to 9 each contained some candidate genes, while most of the candidate genes lay on chromosomes 1 and 8. The gene transcripts selected by CV analysis are listed in Additional file 2: Table S2.

Among the 259 genes, 99 contained more than one nsSNPs. In particular, candidate genes GRMZM2G466563, GRMZM2G070038 and GRMZM2G172320 harbored 13, 12 and 12 nsSNPs with Nonsyn/Syn ratios of 0.40, 0.25 and 0.77, respectively. GRMZM2G466563, a member of calmodulin-binding superfamily, has been demonstrated to be an important signalling component in stress-induced cellular signal transduction pathway [4,31]. GRMZM2G172320, which encodes a keratin-associated protein participated in the formation of rigid and resistant hair shafts in mammalian [32,33], was proven to be involved in water stress signaling pathway [34].

To explore selective constrains and evolutionary divergence of these genes, the Nonsyn/Syn ratio for each candidate gene identified by CV analysis was also investigated using different maize germplasm sets. Among these genes, 46.33% (120 genes) only have nsSNPs in coding region. The Nonsyn/Syn ratios for candidate genes ranged from 0.03 (GRMZM2G104325) to 2.93 (GRMbZM2G071339), with an average of 0.43, of which, 196 genes with the ratios below 0.50 while 8 genes above 1.50. We also calculated the average Nonsyn/Syn ratio of candidate genes using the data from maize HapMap 2, which were collected from a much larger set of germplasm including wild, landrace and improved maize lines [35]. The mean Nonsyn/Syn ratio was 0.46 (0.02 -7.2). Most of the genes (71.4%, 185 out of 259 genes) were under purifying selection with the Nonsyn/Syn ratios below 0.50 (mean value: 0.23). In contrast, only 3.5% of the genes (9 out of 259) were under positive selection with Nonsyn/Syn ratios above 1.5 (mean value: 2.89).

### Variants on chromosome 1 revealed by cluster analysis

**Figure 2:**
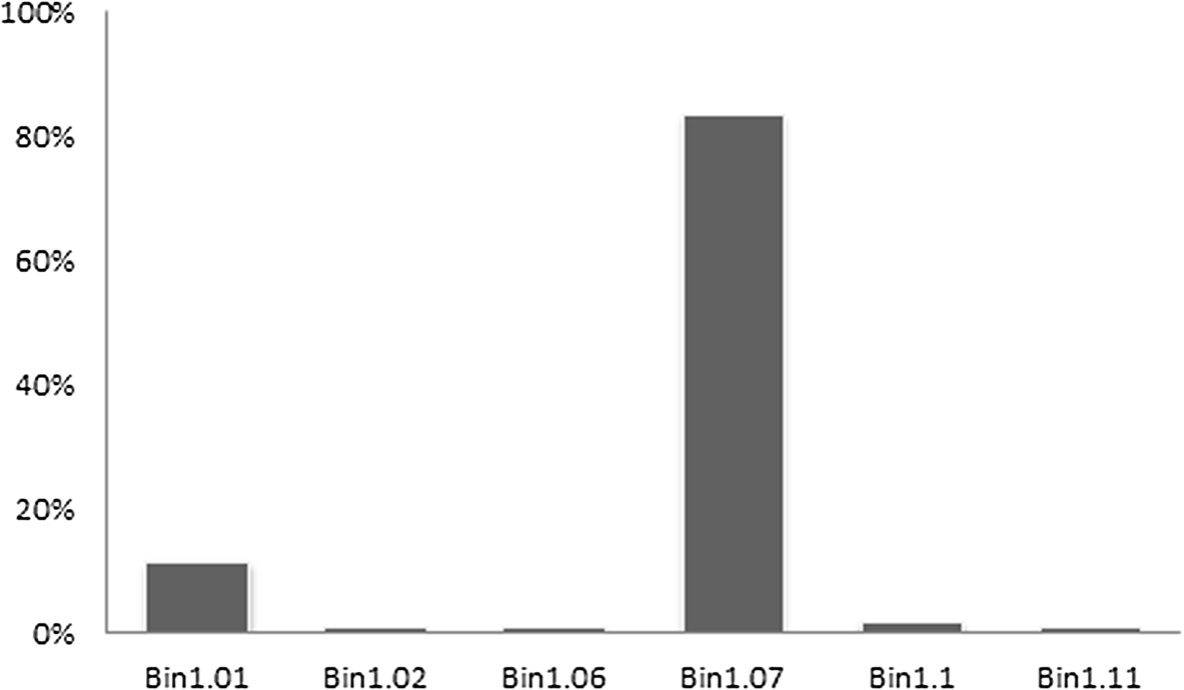
**The cluster regions of candidate nsSNPs for drought tolerance on chromosome 1 identified by cluster analysis.** X-axis represents the bin regions where the clustered nsSNPs located and Y-axis represents the percentages of nsSNPs identified by cluster analysis in each bin region on maize chromosome 1.

To select candidate loci related to drought tolerance, SNP-based cluster analysis proposed by James Silva et al. was carried out with minor modification using all nsSNPs identified with all tested lines [36]. The nsSNPs detected on each chromosome with the tested maize inbreds were used for singular value decomposition (SVD) and Ward’s minimum variance clustering. We used average variant frequency (AVF) with more than 0.8 in extremely drought-tolerant lines but less than 0.1 in extremely drought-sensitive lines to decide the number K of clusters. When the clustered number reached 31, the AVFs on chromosome 1 showed distinct difference between the two groups. The AVF values were 0.010, 0.067 and 0 for the three drought sensitive inbreds, Ye478, Ji853 and B73, while they were 1, 1 and 0.837 for the three drought tolerant inbreds, LX9801, Qi319 and Tie7922, respectively. When the cluster number was less than 31, the drought-tolerant inbred Qi319 had a lower AVF value (less than 0.3) and the drought-susceptive inbred Ji853 had a modest AVF value (close to 0.50). Therefore, the 104 nsSNPs grouped in single cluster 31 on chromosome 1 were selected to represent candidate loci related to drought tolerance. A total of 41 candidate genes (44 transcripts), which were associated with the clustered SNPs, are summarized in Additional file 2: Table S2. Comparing the physical positions with chromosome bin regions of candidate nsSNPs for drought tolerance, we found that 83.65% of candidate nsSNPs were clustered in bin 1.07 (Figure 2), and these nsSNPs were related to genes involved in ABA and cytokinin catabolism, stress signal conduction and redox reaction.

**Figure 3:**
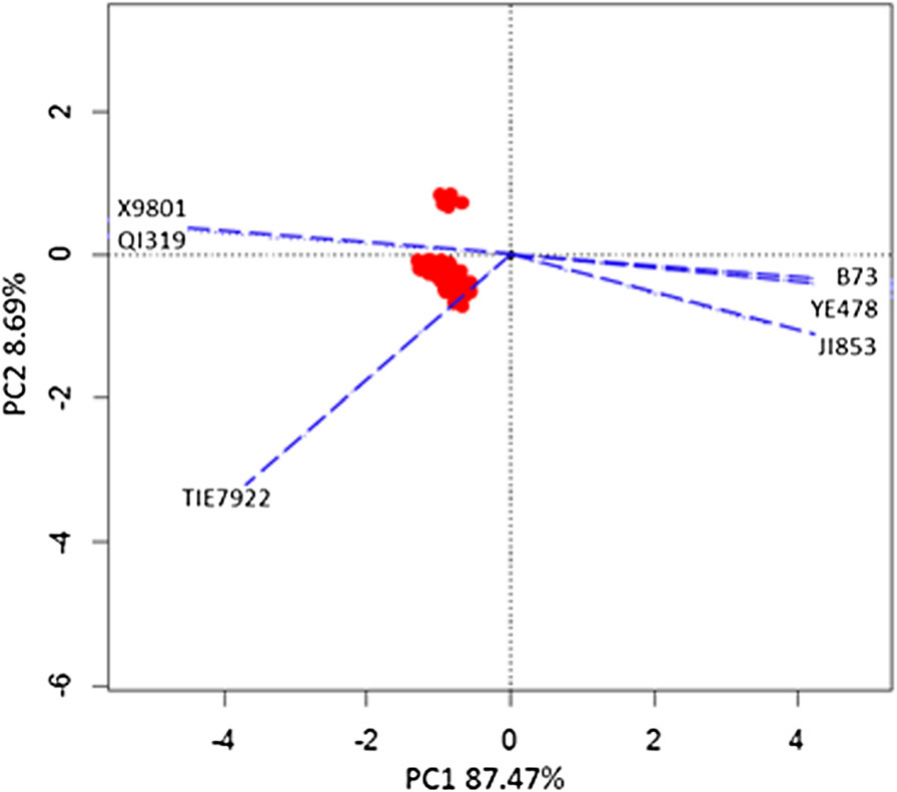
**Biplot display of chosen variants on chromosome 1 in three extremely drought tolerance inbreds (LX9801, Qi319 and Tie7922) and three extremely drought sensitive inbreds (Ye478, Ji853 and B73).** The clustered nsSNPs on chromosome 1 were selected to make the Biplot by transforming the nsSNPs into a (0, 1) matrix. Then the Singular Value Decomposition (SVD) was applied to the matrix with V matrix (for nsSNPs) and G matrix (for materials) returned. The first two vectors of each matrix were used to make X-axis and Y-axis. The blue dotted lines indicate the vectors of the six inbred lines and red round dots represent the chosen variants on chromosome 1.

Biplot was created using the clustered nsSNPs to display the relationships between drought-susceptive inbreds and the candidate nsSNPs. Figure 3 showed the biplot of variants on chromosome 1. Six inbred lines could be divided into two groups using the first and second eigenvectors, which is in accordance with their drought characteristics. The three extremely drought sensitive lines were located around the same region while the drought tolerant lines LX9801 and Qi319 located in the opposite direction of the drought sensitive lines.

### Comparison of candidate genes with previously identified QTL/genes

**Figure 4:**
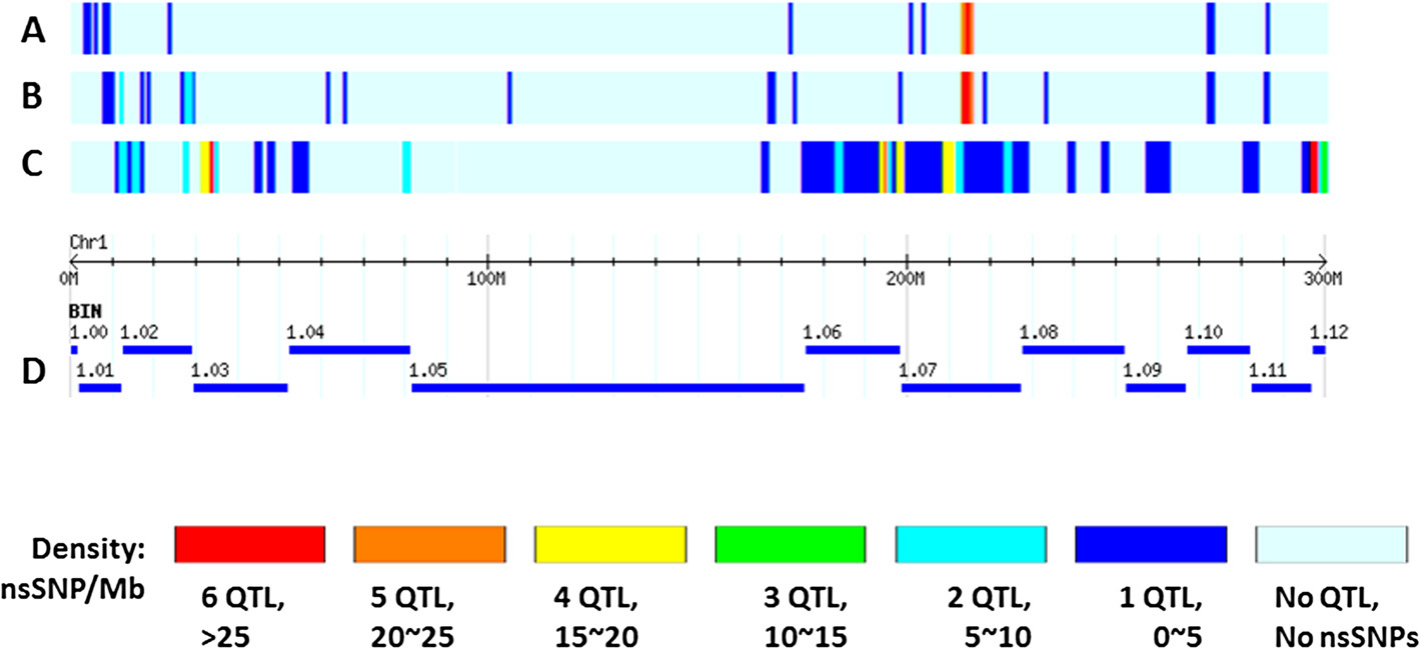
**The densities of candidate nsSNPs by both CV and cluster analyses and reported QTL on chromosome 1 for drought tolerance.** The densities of candidate nsSNPs identified by cluster analysis **(A)** and CV analysis **(B)** and reported QTL **(C)** on chromosome 1 for drought tolerance. **(D)** represents genetic distances and bin regions on chromosome 1.

Both of the CV and cluster analyses successfully identified candidate genes for drought tolerance. A total of 524 nsSNPs were identified by two methods, among which, 79 common variants associated with 28 genes were detected by both methods (Figure 4A and B), which account for 10.8% and 68.3% of the candidate genes revealed by CV strategy and cluster analysis, respectively. More interestingly, we found 77 out of the 79 common variants were clustered in bin 1.07 (Figure 4A and B). In addition, we compared the candidate genes with 48 QTL for drought tolerance on chromosome 1 retrieved from Gramene database ( http://www.gramene.org/) and nine published research articles using different mapping populations and algorithms [5,37-43]. Of the 48 QTL, one for female flowering time [43], two for grain yield [37,39], one for ear number [37], one for stressed-leaf ABA content [44] and one for ASI (anthesis-silking interval) [5] were detected in bin 1.07. The distribution of reported QTL and candidate nsSNPs on chromosome 1 are shown in Figure 4C. The QTL explained relatively high proportions of phenotypic variation (9%-15%). The 26 candidate genes identified by cluster analysis shared the same chromosomal region in bin 1.07 (Figure 2, Figure 4D). These genes were involved in plant hormone regulation, carbonhydrate and sugar metabolism, signalling molecules regulation, redox reaction and acclimation of photosynthesis to environment.

Among the candidate genes identified in bin 1.07, cytochrome P450 (GRMZM2G092823) encodes a key enzyme in ABA catabolism and plays a major regulatory role in controlling the level of ABA in plants [45]. GRMZM2G090264 is a Type-A Arabidopsis response regulator (ARR), which is rapidly induced by cytokinin and is a partially redundant negative regulator of cytokinin signaling [46]. GRMZM2G163437 encodes a subunit of ADP-glucose pyrophosphorylase, which is a key enzyme of the starch biosynthesis pathway [47]. GRMZM2G179063 is glucosyltransferase involved in glucuronoxylan biosynthesis and drought tolerance in Arabidopsis [48]. The putative calmodulin-binding protein (GRMZM2G466563) and leucine-rich repeat receptor-like protein kinase family protein (GRMZM2G428554) play important roles in signal transduction and drought response [49,50]. Besides, induction of peroxidase is a common feature of all the stress treatments [51], and GRMZM2G320269, a peroxidase 27 precursor, maybe involved in the stress response.

### GO enrichment analysis of selected candidate genes

GO based functional enrichment analysis of drought-tolerant candidate genes was performed by the web-based tools AgriGO (Go analysis toolkit and database for agriculture community) ( http://bioinfo.cau.edu.cn/agriGO/index.php) and AgBase ( http://www.agbase.msstate.edu/). The results revealed that 35 GO terms showed significant differences between the candidate genes and all the B73 genes pre-computated as background reference, including 19 GO terms (Additional file 3: Figure S1) involved in biological processes and 16 GO terms (Additional file 4: Figure S2) involved in cellular components. There was no GO term in the category of molecular function. The most enriched terms of biological process ontology were development- and cellular response-related, such as developmental process (GO: 0032502), multicellular organismal development (GO: 0007275), anatomical structure development (GO: 0048856), system development (GO: 0048731), response to biotic stimulus (GO: 0009607), cellular response to chemical stimulus (GO: 0070887) and response to other organisms (GO: 0051707). On the other hand, there was also a significant difference in negative regulation of biological process (GO: 0048519), negative regulation of cellular process (GO: 0048523) and chromatin modification (GO: 0016568). To the cellular component ontology, candidate genes were enriched in membrane and vesicle related cellular component including the membrane-bounded organelle (GO: 0043227), intracellular membrane-bounded organelle (GO: 0043231), cytoplasmic part (GO: 0044444), vesicle (GO: 0031982), plastid (GO: 0009536) and membrane-bounded vesicle (GO: 0031988).

**Figure 5:**
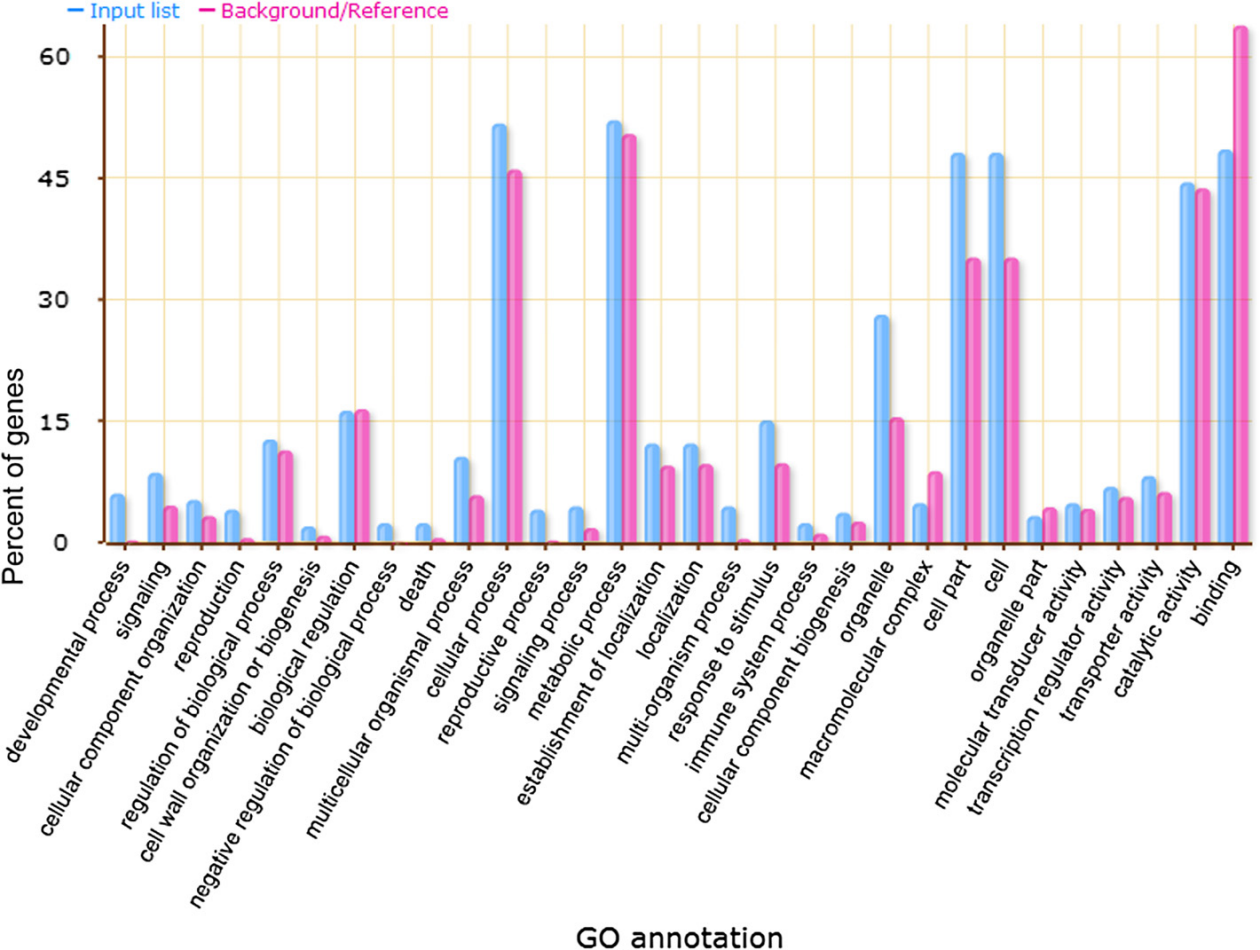
**Flash bar chart of over represented terms for drought-tolerant candidate genes in biological process category.** The Y-axis is the percentage for the input genes in different GO terms calculated by the number of genes mapped to the GO terms divided by the number of all input genes. The same calculation was applied to the reference list to generate its percentage. These two lists are represented using different custom colors. The X -axis is the definition of GO terms.

A detailed comparison of biological process groups involved in drought responses to background is provided in Figure 5. With the biological process ontology, developmental process (GO: 0032502) and signalling (GO: 0023052), multicellular organismal process (GO: 0032501) and response to stimulus (GO: 0050896) were enriched for the drought response candidate genes. Meanwhile, negative regulation of biological process (GO: 0048519) and death (GO: 0016265) also showed a relatively high rate than the all given genes from reference genome B73 as background.

### Validation of candidate genes

**Figure 6:**
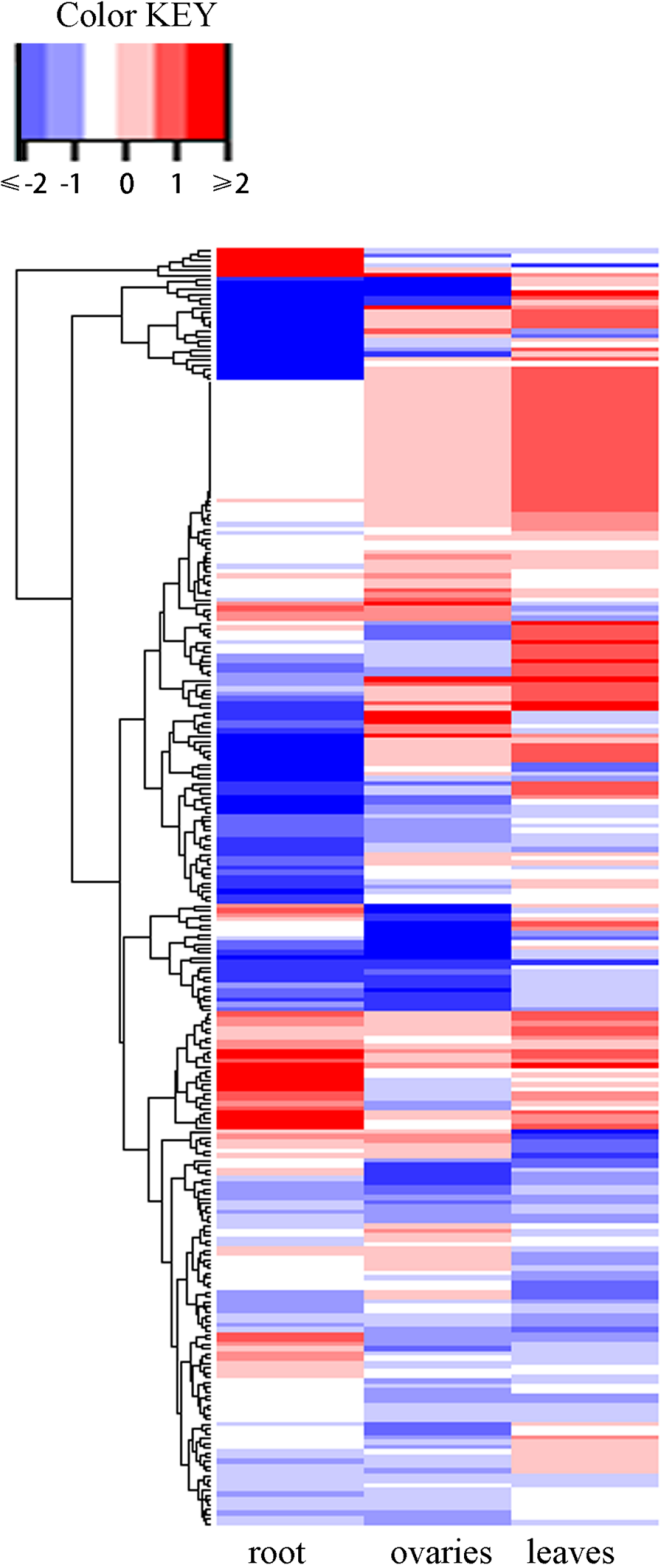
**Clustering of candidate genes according to their changed expression levels in water-stress condition.** The color scale shown on the top left represents the changed gene expression values (Log_2_ fold change) under water-stressed condition. “roots”, “ovaries” and “leaves” column present the tested genes in the roots, ovaries and the basal leaves, respectively. The dose red and blue colors represent up-and down-regulated expression, respectively.

To validate whether the selected candidate genes respond to drought tolerance, we examined expression level changes of 271 candidate genes through transcriptome analysis of the roots from drought tolerant inbred AC7643, and the leaves and ovaries from drought sensitive inbred line B73 under well-watered and water-stressed conditions. The fold changes of candidate genes expression responsive to water stress in ovaries, leaves and roots are displayed in Figures 1D,E and F, respectively. A total of 262 genes revealed by CV and cluster strategy showed change of their expression levels in different water conditions, of which 181 genes (around 70%) changed significantly (P < 0.05) and 77 genes had a fold change of more than two in ovaries, leaves or roots. In drought tolerant inbred AC7643, 177 genes displayed significantly different expression in roots under two water treatments, including 43 up-regulated genes and 134 down-regulated genes. The expression level of aserine/threonine-protein kinase family member (GRMZM2G179789) substantially changed, with a 7-fold-increase under water-stress condition. A hypothetical protein (GRMZM2G050741) exhibited a more than 9-fold decrease in expression level under water-stress condition. Although the candidate genes showed different expression characters due to the different tissues and inbreds used for RNA sequencing, the relatively high rate of genes significantly altered their expression levels under water-stress condition, which indicated these candidate genes identified by CV and cluster strategies were associated with drought tolerance. Expression level difference of candidate genes in ovaries, leaves and roots under two water treatments and the expression change based hierarchical clustering are shown in heat map with different colors representing relative mRNA expression (Figure 6).

### Validation of SNPs

**Figure 7:**
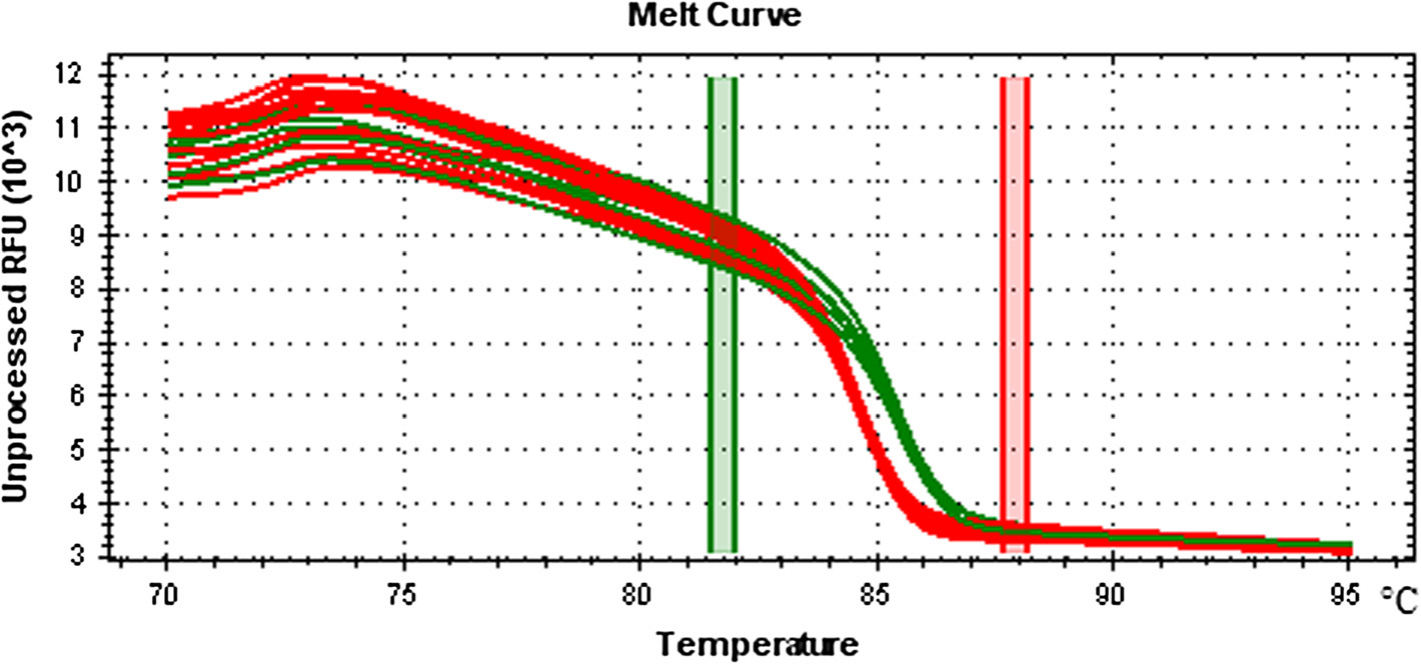
**High resolution melting analysis (HRM) of PCR amplicons for gene GRMZM2G467339 in 16 maize inbreds.** Red and green curves indicate SNP loci A and G, respectively.

To verify the accuracy of SNPs, comparison of the 46,556 loci identified from Illumina SNP50K Chip and SNPs called from 12 resequencing inbreds were performed. The results indicated that more than 99% of SNPs were in accordance with the physical positions and genotypes. The SNP discordant rates between two datasets were presented in Additional file 1: Table S1. In addition, all the 16 inbred lines were used for SNP validation through PCR amplification and HRM validation. Five candidate genes were randomly selected for validation and corresponding five primer pairs were designed (Table 2). The HRM result of PCR amplicons for the candidate gene GRMZM2G467339 is shown in Figure 7. The two groups with SNP locus “A” in red curves and “G” in green curves in 16 inbred lines were distinguished successfully. The sequence of amplicon with the SNP in “A” had a lower melting temperature compared with single base mutation of “G”. The difference in melt temperature indicated the SNP existed in the chosen maize inbred lines. The HRM genotyping results also confirmed that the candidate nsSNPs were consistent with the sequences generated by NGS.

**Table 2:** Information of the primers used for high resolution melting (HRM) analysis

**Primers**	**Target gene (ID)**	**Product size**	**Annealing temperature**	**Primer sequence (5′-3′) (bp)**
		**(bp)**	**(°C)**	
1	GRMZM2G072292	88	62	F: GCAAGCGGGGACATGAGC
				R: TCTTGGAGAAGCCCAGCGA
2	GRMZM2G055844	69	62	F: TATGTCCAGTCAGCGAGAG
				R:GGCTATGTCCACGATCATTG
3	GRMZM2G386229	68	62	F: GAGGCGTTCTACTCCGAG
				R: AGCGACAGGAGACAGTAC
4	GRMZM2G467339	90	59	F: GTATGTCTTAATAGGTATGTCTCA
				R: GTACACCCGATGTTCTTC
5	GRMZM2G109448	70	60	F: GCTGTCTCATCCTCATCG
				R: CCAATCTGTGAAGAAGTGAAG

## Discussion

### Functional and regulatory genes for drought tolerance in maize

Plant roots have the ability to grow toward the direction of high water availability and away from that of high osmolarity (hydrotropism). Xiong et al. searched for phenotypes conferred by drought stress and identified the inhibition of lateral root development by drought stress as an adaptive response to the stress [52]. Ovaries in tissue subjected to drought stress stop growth within 1 to 2 day after pollination [53], and tolerance to water stress in female floral parts has been correlated with yield in maize [54]. Gene expression studies in maize in response to water stress have been conducted in roots [55], seedlings [56], and developing ear and tassel [57]. In the study, transcriptome analysis of leaves, ovaries and roots from drought sensitive inbred and tolerance inbred was thus performed to further validate the candidate genes and elucidate mechanisms for its regulation.

The response of plants to drought stress is very complex and involves lots of genes and pathways related to diverse mechanisms [4,9,58,59]. However, some secondary physiological traits have been investigated as a drought tolerance measurement and some universal genes, such as NAC transcription factors, are involved in abiotic stress response in different varieties and even species [60,61]. This provides us an opportunity to mine important universal drought response genes by assessing the variations capably inducing modification of the protein sequences in maize inbreds with different genetic backgrounds.

In this study, we identified genes involved in plant hormone regulation (especially ABA synthesis and metabolism), carbonhydrate and sugar metabolism, signalling molecules regulation and redox reaction. These genes may function as regulatory protein factors involved in the regulation of signal transduction and gene expression functioning in stress responses [62]. One of the major unresolved issues concerning the genetic architecture of abiotic stress response is whether functional variation arises from variation in core signaling components, such as transcription factor, kinases and phosphatases, or these variations are confined to effector genes, such as biosynthetic enzymes, redox regulators and heat shock proteins [63]. Gene families with essential functions (for example, ubiquitin and cellulose synthase families) in rice tended to have substantially lower Nonsyn/Syn ratios, whereas gene families that functioned in regulatory processes and signal recognition, such as disease resistance family, had higher ratios [64]. In our research, candidate genes with more than 10 nsSNPs involving in stress signaling pathway and functioning as regulators also had higher Nonsyn/Syn ratios, which were consistent with the results in rice.

On the other hand, from an evolutionary viewpoint, more than 70% of the candidate genes were under negative selection with a relatively low average Nonsyn/Syn ratios in both maize inbred lines population and a much larger set of germplasm including wild, landrace and improved maize lines. The result indicated that these genes possess central and essential functions and nonsynonymous mutations impacting on the genes function have been removed by purifying selection [65]. A similar result was observed in *Eucalyptus camaldulensis* seedlings subjected to water stress via transcriptome sequencing [66].

### CV and cluster analyses for mining candidate genes

Recent advances in whole-genome sequencing have allowed identification of candidate genes responsible for abiotic and biotic stresses. Silva et al. used CV and principal component-biplot (PB) selection strategy to exploit whole genome sequences of 13 rice inbred lines and identify nsSNPs and candidate genes for resistance to sheath blight, a disease of worldwide significance [33]. In our study, both CV and cluster analyses successfully identified the candidate genes associated with drought tolerance. Gene expression studies through RNA-seq on ovaries and basal leaves of drought sensitive inbred B73 confirmed that around 80% of the candidate genes showed decreased or increased expression under water-stress condition [17]. Moreover, transcriptome analysis conducted on the roots of drought tolerant inbred AC7643 validated 65.7% of candidate genes displayed significantly different expression under water-stressed conditions, including 44 up-regulated genes and 134 down-regulated genes. Interestingly, the candidate genes identified by CV analysis showed more significant and severe change in expression level, indicating that CV analysis might be more efficient than clustering. However, from methodology perspective, the procedure of CV analysis is somewhat tedious while cluster analysis is more systematic as described by Silva et al. [36]. Besides, cluster analysis has another advantage that the candidate loci identified can be clustered in some chromosomal regions. In our analysis, the majority of candidate nsSNPs detected by cluster analysis were located in bin 1.07, accounting for 83.65% of the total candidate nsSNPs. Compared with the reported QTL for drought tolerance, this chromosomal region (bin 1.07) harbored important QTL involving in flowering time and grain yield under water-stress condition, suggesting that cluster analysis was credible and successful in mining candidate genes for the target traits in our study. Moreover, more than 10% of the candidate genes could be identified by both methods, most of which were clustered on chromosome 1 (bin1.07). For a large number of clusters, candidate SNPs identified by both methods were almost indistinguishable [36].

### Functions of SNPs in different genomic regions

SNPs were very commonly used for association studies to identify genes or genetic regions contributing to complex traits [67,68]. From these genome-wide researches, SNPs could be identified in almost all genomic regions to explain variation of phenotypic traits to various degrees. From the point of view of molecular level, functional SNPs can affect the phenotype by interfering both transcription level and protein synthesis [69]. It has been long considered that the SNPs on protein-coding sequences have potential effects on gene function, especially the nsSNPs that could lead to amino acid residue changes and altered functional or structural properties of the protein. Although the non-coding SNPs could not cause any amino acid change, they may affect transcription factor binding sites, splice sites and other functional sites in transcriptional level. In maize, 21% of the SNPs in HapMap 2 were associated with a genic region [35], which suggested that the polymorphisms in coding sequences were less than non-coding areas. Furthermore, Li et al. analyzed genic and non-genic contributions to natural variation of quantitative traits in maize and revealed that 79% of the explained variation could be attributed to trait-associated SNPs located in genes or within 5 kb uptream of genes [70]. This indicates that variations in genic and promotor regions would be more important in genetic resolution of complex traits. The less in numbers but more significant in terms of functions has made the nsSNPs an ideal marker type in complex trait association analysis. More than 200 genes with selected nsSNPs for resistance to sheath blight disease were detected in rice by whole-genome sequencing. In the study, we focus on the nsSNPs and drought associated candidate genes within nsSNPs were successfully detected by comparative analysis of different maize inbred lines.

### Genetic resources for drought tolerance in maize

Maize is an important crop for food, feed, forage, and fuel across tropical and temperate areas of the world. Diversity studies at genetic, molecular, and functional levels have revealed that tropical maize germplasms, landraces and wild relatives harbor a significantly wider range of genetic variation. Landraces from dry habitats have been used successfully in breeding for water limited environments, and wild species and progenitors of our cultivated crops were always on the agenda as possible donors for drought tolerance [71,72].

From an evolutionary perspective, drought is an important abiotic stress that influences yield with strong interactions between genes and environment [73], which was also an important evolutionary force responsible for population diversification in some species [74]. Plants exhibit morphological and physiological adaptations to cope with environmental stresses. However, evidence for selection (natural or artificial) of drought tolerance has rarely been examined in maize. Many researches have indicated that, the ancestor of maize, teosinte, is a drought tolerant grass while domesticated landraces and inbred lines have differentiated in drought tolerance. As a result, some landraces and inbred lines are drought tolerant while others are drought susceptible. Back to the process of domestication, drought tolerance might be selected together with plant productivity in farming practice, and comparing with wild type, domesticated plants reduced defense ability exposure to biotic and abiotic stresses (which was identified in sunflower but has not been reported in maize) [75,76]. Therefore, identifying more drought tolerance genes and exploring the nature of drought tolerance may open new avenues for their use in maize improvement.

The advent of whole genomics technologies provides necessary tools for identifying the key gene networks that respond to drought stress [77]. Based on all available knowledge for the traits related to yield and drought tolerance, randomly dispersed QTL, trans-genes or both can be accumulated into elite genotypes through “breeding by design” [15,78]. Better understanding of the genetic bases of the secondary drought tolerance traits and analysis of allelic variation at the corresponding loci would enable the breeders to design new ideotype crops.

## Conclusions

A total of 524 nsSNPs were selected by CV analysis and clustering using B73 reference genome and whole-genome resequencing of 15 maize inbreds with various drought characteristics. Two hundred seventy one drought-tolerant candidate genes corresponding to the candidate nsSNPs were identified, which involved in a variety of physiological and metabolic pathways in response to the water stress. GO based function analysis and comparison of candidate genes with reported drought associated QTL indicated that these candidate genes were notably associated with drought tolerance. Furthermore, about 70% of candidate genes showed significantly expression change under two water conditions by transcriptome analysis of fertilized ovaries, basal leaves and roots. Two methods used in the study are efficient approaches for detecting candidate genes underlying complex traits, including drought tolerance. Results from this study also provide a foundation for future basic research and marker-assisted breeding for improving drought tolerance in maize.

## Methods

### Plant materials and DNA extraction

A total of 16 maize inbred lines were selected based on their drought responses identified in our previous experiments [61] and other reports [24-26] based on selection criteria such as grain yield, anthesis-silking interval and leaf senescence under well-watered and water-stressed environments (Table 3). Among them, maize inbred lines B73, Ye478 and Ji853 were extremely drought-sensitive, while LX9801, Qi319 and Tie7922 were extremely drought-tolerant. Besides, 10 maize inbred lines with moderate drought sensitive tolerance were also used in the study. These materials were chosen from different heterotic groups, Stiff Stalk (SS) and non-Stiff Stalk (NSS) [70,71] and heterotic group containing tropical or subtropical maize inbreds (TST). Genomic DNA was extracted from 2-week old seedlings using CTAB method.

**Table 3:** Maize inbred lines used in the study

**Name**	**Pedigree**	**Origin**	**Drought tolerance characteristics**	**Selection criteria**	**References**	**Heterotic groups**	**Adaptation**
AC7643	Unknown	CIMMYT	Moderate tolerance	NDVI, ASI, LS, CC, RC, GY, GYC	[5,61]	TST	Tropical/subtropical
CML206	[EV7992#/EVPO44SRBC3]#BF37SR-2-3SR-2-4-3-BB	CIMMYT	Moderate sensitive	ASI, LS, GY, GYC, PH	[25]	TST	Tropical/subtropical
Ye478	U8112/Shen5003	China	Extremely sensitive	ASI, LS, GY, GYC, PH, RWC	[25,26]	SS	Temperate
Dan598	(Dan340/Danhuang11)/(Danhuang02/Dan599)	China	Moderate tolerant	ASI, LS, GY, GYC, PH	[25]	NSS	Temperate
Si287	444/255	China	Moderate tolerant	ASI, LS, GY, GYC, PH	[25]	SS	Temperate
Ji853	(Huangzao4/Zi330)/Zi330	China	Extremely sensitive	ASI, RWC, MDA, EC	[27]	NSS	Temperate
LX9801	Ye502 × H21	China	Extremely tolerant	ASI, LS, GY, GYC, PH	[25]	SS	Temperate
Qi319	American hybrid P78599	China	Extremely tolerant	ASI, LS, GY, GYC, PH	[25]	NSS	Temperate
Tie7922	American hybrid P3382	China	Extremely tolerant	ASI, LS, GY, GYC, PH	[25]	SS	Temperate
Han21	American hybrid P78599	China	Moderate tolerant	ASI, LS, GY, GYC, PH	[25]	NSS	Temperate
Zheng22	(Duqing/E28)/Lujiukuan	China	Moderate sensitive	ASI, LS, GY, GYC, PH	[25]	SS	Temperate
Ji419	Si419/(B68HT/Mo17)	China	Moderate sensitive	ASI, LS, GY, GYC, PH	[25]	NSS	Temperate
ES40	Landrace Linshuidadudu from Sichuan	China	Moderate sensitive	ASI, PH, EC, GY	[24]	SS	Subtropical
81565	(Huobai/Jing03)/(S2/Heibai94)	China	Moderate tolerant	ASI, PH, EC, GY	[24]	TST	Tropical/subtropical
X178	Selected from an introduced hybrid	China	Moderate tolerant	ASI, PH, EC, GY	[24]	SS	Temperate
B73	BSSS	China	Extremely sensitive	RWC, LAI, RWC	[28,29]	SS	Temperate

*SS* Stiff stalk heterotic group with representativeness of B73. *NSS* Non stiff stalk heterotic group. *TST* heterotic group containing tropical or subtropical maize inbreds. *NDVI* normalized difference vegetation index. *ASI* anthesis-silking interval. *LS* leaf senescence. *CC* chlorophyll content. *RC* root capacitance. *GY* grain yield. *GYC* grain yield components. *PH* plant height. *RWC* relative water content. *MDA* maleic dialdehyde. *EC* electric conductivity. *LAI* leaf area index.

### Maize genome sequencing, SNP calling and nsSNP identification

Sequences were generated for maize lines while paired-end libraries were constructed according to the Illumina manufacturer’s instructions. Whole-genome resequencing was performed on Illumina Hiseq 2000 platform for 15 maize inbreds and a total of 4.6 billion (407 gigabases) sequence reads were aligned against the maize B73 reference genome ( http://www.maizesequence.org, Release 5b) using SOAP 2 [22] ( http://soap.genomics.org.cn/) which is a widely used reads alignment tool. There were around 1.8 billion reads were uniquely mapped onto B73 reference genome, with average of 0.12 billion reads for each maize inbred (Additional file 1: Table S1). SNP calling and validation were performed as Chia et al. [35]. Sequencing and SNP calling were carried out at BGI (Shenzhen, China). The nsSNPs within the genes were filtered using ANNOVAR tool [23] that can be used to functionally annotate a list of genetic variants including intronic, exonic, intergenic, 5’/3’-UTR, splicing site and upstream/downstream variants. Promotor sequences were determined at 2 Kb upstream of transcription initiation site.

### Identification of nsSNPs in candidate drought tolerance genes

Common variants (CV) analysis and SNP based cluster analysis proposed by James Silva et.al [36], with minor modification, were used separately to identify nsSNPs and their associated candidate genes for maize drought tolerance. The CV analysis for filtering candidate genes was taken as following steps: 1) screening SNPs which were common within the two groups containing three extremely drought-tolerant maize inbreds (LX9801, Qi319 and Tie7922) and three drought-sensitive inbreds (B73, Ye478 and Ji853), respectively; 2) selecting candidate nsSNPs that were different between two groups for drought tolerance; 3) identifying associated candidate genes for maize drought tolerance using the selected nsSNPs.

To identify efficiently nsSNPs related to drought tolerance, SNP based cluster analysis was also carried out using all nsSNPs detected from all tested lines. The strategy includes the following steps. 1) Remove common variants across the tested materials and transform the remaining nsSNPs into a (0, 1) matrix. At each locus, variant frequency was denoted by “0” if the allele was the same with that in B73 reference genome representing drought-sensitive line; otherwise it was denoted by “1”. 2) Singular Value Decomposition (SVD) was applied to standardize variant frequencies in the matrix. The SVD procedure returned three matrixes, V (for nsSNPs), D (diagonal containing eigenvalues) and G (for materials). Ward’s minimum variance clustering was performed using V matrix in SAS software (Release 9.3; SAS Institute, Cary, NC, USA). 3) For each cluster identified in step 2, the average values of variant frequencies were calculated for the 16 maize inbreds. The single cluster with AVF > 0.8 in extremely drought-tolerant lines but < 0.1 in extremely drought-sensitive lines were selected for each chromosome. 4) Screen significant nsSNPs based on step 3, and identify the corresponding candidate genes for drought tolerance. 5) Create GGEbiplot display using clustered nsSNPs through GGEBiplotGUI package of R program.

### Gene ontology (GO) analysis of selected candidate genes

Candidate genes were submitted to AgriGO (Go analysis toolkit and database for agriculture community) ( http://bioinfo.cau.edu.cn/agriGO/index.php) and AgBase ( http://www.agbase.msstate.edu/) for gene ontology analysis [79]. Singular enrichment analysis (SEA) was used to select enrichment GO terms ( http://bioinfo.cau.edu.cn/agriGO/analysis.php) with the maize reference genome B73 as background (Maizesequence, version: 5b). The over represented terms in three categories, biological process, cellular component and molecular function, were filtered by statistical information including Fisher’s exact test and the Bonferroni for multi-test adjustment method [79].

### Validation of candidate genes using RNA-seq data

To further validate the candidate genes for drought tolerance revealed by CV and SNP based cluster analysis, the expression level of candidate genes under two water conditions was evaluated using transcriptome analysis of drought tolerant inbred AC7643 and available RNA-seq data of drought sensitive inbred B73 ( http://www.ncbi.nlm.nih.gov/sra/).

For transcriptome analysis, the seeds of maize inbred AC7643 were surface-sterilized and grew in the same nutrient solution and environment condition as reported [80]. At the three-leaf-stage, 20% PEG were subjected for 24 h and the roots of inbred AC7643 under well-watered and drought-stressed conditions were sampled for RNA extraction separately using TRIzol® reagent (Invitrogen, USA), RNA sequencing were performed at BGI-Shenzhen (Shenzhen, China) using Illumina deep sequencing according to the manufacturer’s instructions.

The RNA-seq data obtained using Illumina deep sequencing from leaf meristem and pollinated ovaries of drought sensitive inbred B73 under well-watered and water-stressed conditions were downloaded from the publicly available databases SRA project (SRP014792) ( http://www.ncbi.nlm.nih.gov/sra/). NCBI SRA toolkit was used for data format exchanging [81]. All raw reads were then applied in the FASTX toolkit ( http://hannonlab.cshl.edu/fastx_toolkit/) for reads quality control prior to mapping. The fastx_clipper and fastx_artifacts_filter programs were used to remove Illumina adapter sequences and artifactual sequences such as homopolymeric sequences. Then low quality reads with length shorter than 30 bp or less than 33 of Phred score were discarded using the fastq_quality_trimmer. Qualified RNA-seq reads were mapped to the maize B73 reference genome ( http://ftp.maizesequence.org/release-5b/) with known transcripts and annotation ( http://ftp.maizesequence.org/) using programs Bowtie2 (version2.0.2) and TopHat (version 2.0.6) [82,83]. HTSeq-DEseq workflow was used for differential expression analysis [84]. A false discovery rate of 0.05 after Benjamini-Hochberg correction for multiple tests was applied. The expression heat map for candidate tolerance genes was made by the R ggplot2 package.

### SNP validation using gene chip and HRM

In our previous research, 12 of 15 resequencing samples were genotyped with Illumina Maize SNP50 chip, from which, 46,556 high-quality SNPs were selected and then their probe sequences were mapped to the B73 reference to get the exact physical positions. To verify the accuracy of SNPs, comparison of chip-based genotyping and SNP calling from 12 resequencing inbreds were conducted based on their physical positions. Besides, five primer pairs were designed for the five candidate genes containing target nsSNPs (Table 2), and genomic DNA of the 16 tested maize inbred lines were chosen as the templates. PCR reactions were performed on Bio-Rad CFX96 real-time PCR detection system (Bio-Rad, Inc., Hercules, CA). The reaction volume and cycling conditions were followed by the SsoFastTMEvaGreensupermix (Bio-Rad) manual. All samples were amplified in duplicate reactions and together with a non-template control. HRM curve data was analyzed using the manufacturer’s software.

## Competing interests

The authors declare that they have no competing interests.

## Authors’ contributions

YL, YX and TR designed this study and participated in its coordination. GP, QT, SG and HL provided samples required for sequencing. GZ, XG performed genome sequencing and SNP calling. JX, YL and YY performed the data analysis. YY, QW, FW and JW prepared the samples for sequencing and conducted the experiments. JX, YL and MC wrote the manuscript. All authors read and approved the final manuscript.

## Supplementary Material

Additional file 1: Table S1 The detailed resequencing information and SNP discordant rate between chip-based genotyping and resequencing. Click here for file

Additional file 2: Table S2 The transcripts identified by common variants (CV) and cluster analyses, A = alanine, C = cysteine, D = aspartic acid, E = glutanic acid, F = phenylalanine, G = glycine, H = hisitidine, I = isoleucine, K = lysine, L = leucine, N = asparagine, M = methionine, P = proline, Q = glutamine, R = arginine, S = serine, T = threonine, W = tryptophan, Y = tyrosine, V = valined. Click here for file

Additional file 3: Figure S1 Hierarchical tree graph of overrepresented GO terms in biological process category generated by singular enrichment analysis. Boxes in the graph represent GO terms labeled by their GO ID, term definition and statistical information. The significant (adjusted P < = 0.05) and non-significant terms are marked with color and white boxes, respectively. The diagram, the degree of color saturation of a box is positively correlated to the enrichment level of the term. Solid, dashed, and dotted lines represent two, one and zero enriched terms at both ends connected by the line, respectively. The rank direction of the graph is set to from top to bottom. Click here for file

Additional file 4: Figure S2 Hierarchical tree graph of overrepresented GO terms in cellular component category generated by singular enrichment analysis. Boxes in the graph represent GO terms labeled by their GO ID, term definition and statistical information. The significant (adjusted P < = 0.05) and non-significant terms are marked with color and white boxes, respectively. The diagram, the degree of color saturation of a box is positively correlated to the enrichment level of the term. Solid, dashed, and dotted lines represent two, one and zero enriched terms at both ends connected by the line, respectively. The rank direction of the graph is set to from top to bottom. Click here for file
