# Supplementary figures and images for: Identification of candidate genes for drought tolerance by whole-genome resequencing in maize
Source: BMC Plant Biol. 2014 Apr 1;14:83. doi: 10.1186/1471-2229-14-83 (PMC4021222; doi:10.1186/1471-2229-14-83)

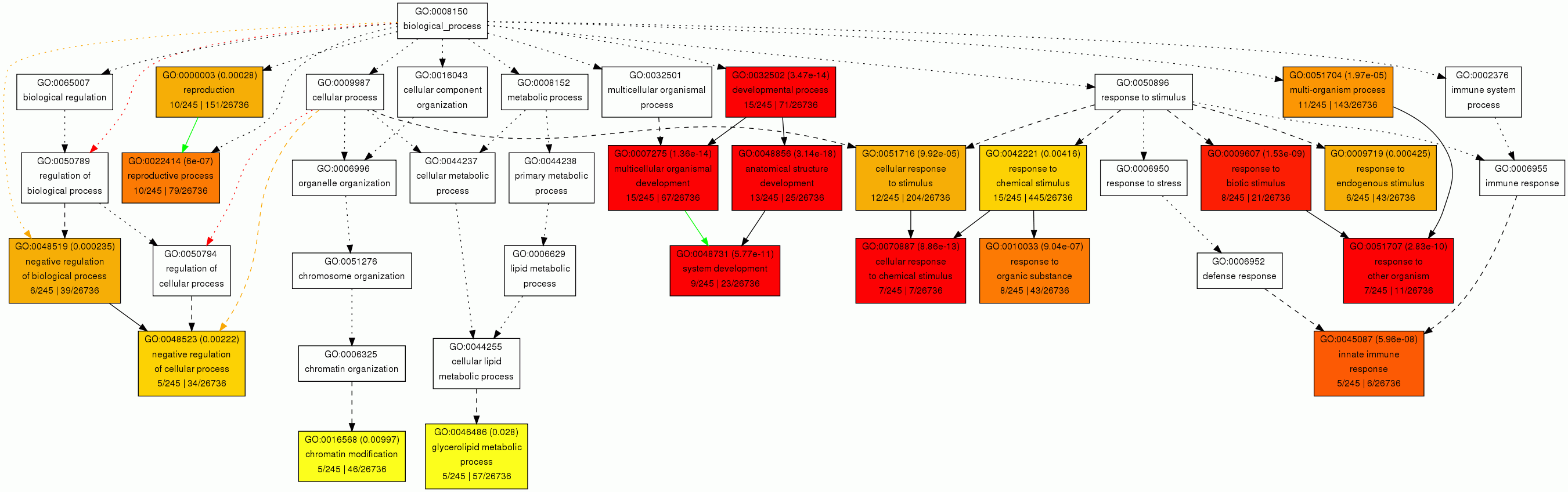

Supplement: Additional file 2: Table S2 — The transcripts identified by common variants (CV) and cluster analyses, A = alanine, C = cysteine, D = aspartic acid, E = glutanic acid, F = phenylalanine, G = glycine, H = hisitidine, I = isoleucine, K = lysine, L = leucine, N = asparagine, M = methionine, P = proline, Q = glutamine, R = arginine, S = serine, T = threonine, W = tryptophan, Y = tyrosine, V = valined. [file 1471-2229-14-83-S2.gif]

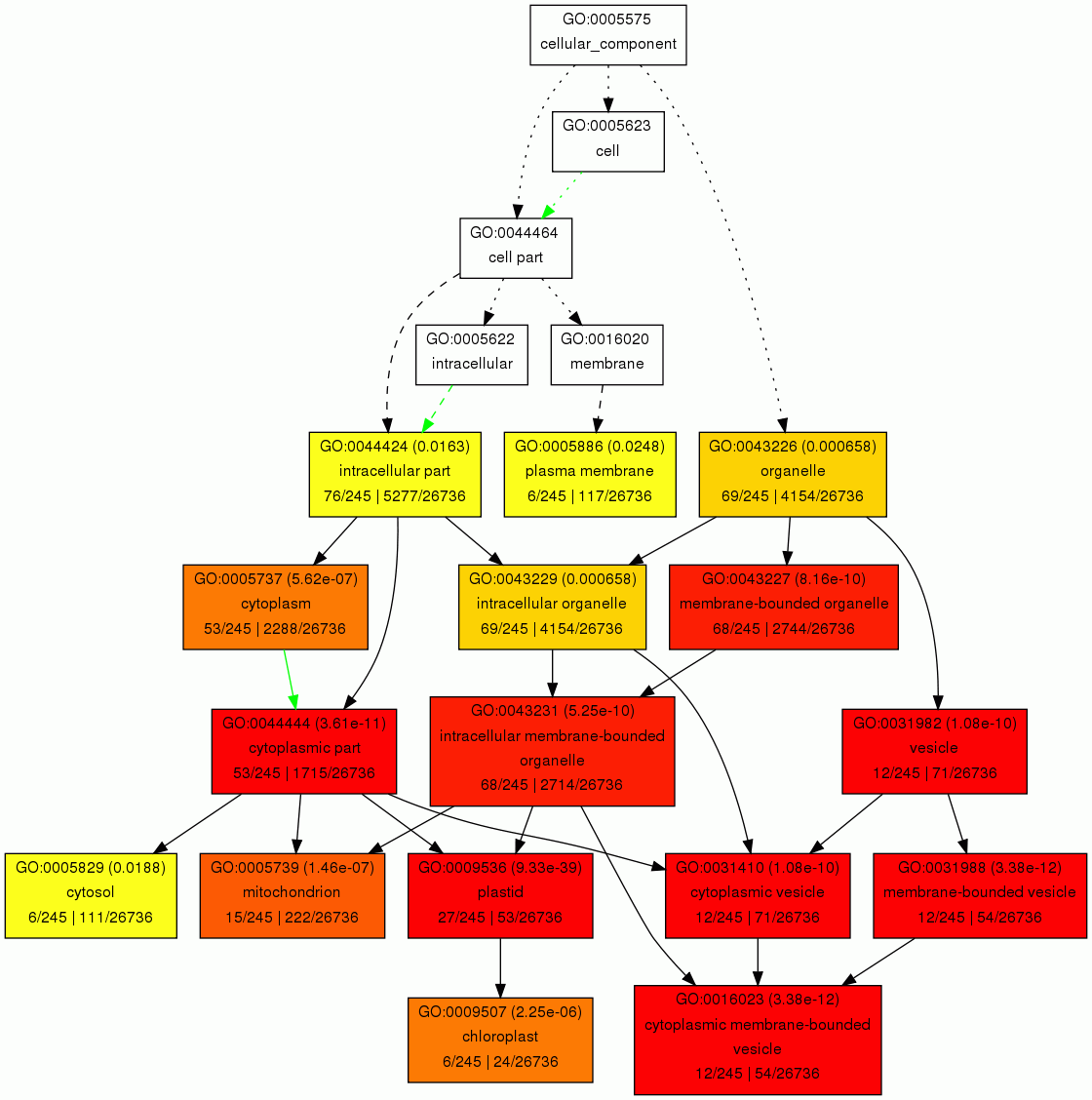

Supplement: Additional file 3: Figure S1 — Hierarchical tree graph of overrepresented GO terms in biological process category generated by singular enrichment analysis. Boxes in the graph represent GO terms labeled by their GO ID, term definition and statistical information. The significant (adjusted P < = 0.05) and non-significant terms are marked with color and white boxes, respectively. The diagram, the degree of color saturation of a box is positively correlated to the enrichment level of the term. Solid, dashed, and dotted lines represent two, one and zero enriched terms at both ends connected by the line, respectively. The rank direction of the graph is set to from top to bottom. [file 1471-2229-14-83-S3.gif]
